# Effects of mesenchymal stem cells on solid tumor metastasis in experimental cancer models: a systematic review and meta-analysis

**DOI:** 10.1186/s12967-018-1484-9

**Published:** 2018-04-27

**Authors:** Jing-Huan Li, Wen-Shuai Fan, Mi-Mi Wang, Yan-Hong Wang, Zheng-Gang Ren

**Affiliations:** 1 Key Laboratory of Carcinogenesis and Cancer Invasion, Ministry of Education, Shanghai, 200032 China; 2 Liver Cancer Institute, Zhongshan Hospital, Fudan University, Shanghai, 200032 China; 3 Ruijin Hospital, Shanghai Jiaotong University School of Medicine, Shanghai, 200025 China; 4 Department of Orthopedics, Zhongshan Hospital, Fudan University, Shanghai, 200032 China

**Keywords:** Neoplasm metastasis, Mesenchymal stem cells, Animal model, Meta-analysis

## Abstract

**Background:**

It has been reported mesenchymal stem cells (MSCs) are recruited to and become integral parts of the tumor microenvironment. MSCs might have an active role in solid tumor progression, especially cancer metastasis. However, the contribution of MSCs in the process of cancer metastasis is still controversial. In this review, we performed a meta-analysis on the effects of MSCs administration on cancer metastasis based on published preclinical studies.

**Methods:**

The PRISMA guidelines were used. A total of 42 publications met the inclusion criteria. Outcome data on the incidence and the number of cancer metastasis as well as study characteristics were extracted. Quality of the studies was assessed according to SYRCLE Risk of Bias tool. Random-effects meta-analysis was used to pool estimates.

**Results:**

Of the 42 studies included, 32 reported that MSCs administration promoted outcome events (numbers or incidences of cancer metastasis), and 39 reported data suitable for meta-analysis. The median effect size (RR) was 2.04 for the incidence of cancer metastasis (95% CI 1.57–2.65, I^2^ = 21%), and the median effect size (SMD) was 1.23 for the number of cancer metastasis (95% CI 0.43–2.03, I^2^ = 89%). Heterogeneity was observed, with the greater impact based on study length and different ways of metastasis measurement and MSCs administration.

**Conclusion:**

Our results suggested MSCs administration increased the number and the incidence of cancer metastasis in experimental cancer models. High heterogeneity and poor reported risk of bias limit the quality of these findings. Further preclinical studies with better design and adequate reporting are still needed.

**Electronic supplementary material:**

The online version of this article (10.1186/s12967-018-1484-9) contains supplementary material, which is available to authorized users.

## Background

Distant metastasis is a major feature of cancer cells, which is responsible for most cancer-associated mortality [1]. The biology of the cancer cells plays an important role in cancer metastasis. However, more and more evidence supports the role of cancer-associated stroma in cancer metastasis and poor clinical outcomes [2–4]. And therapeutic candidates that targeted stromal members are widely explored to improve outcomes of cancer treatment [3].

Mesenchymal stem cells (MSCs) have been successfully isolated from several primary solid tumors, such as ovarian cancer, breast cancer, gastric cancer, and osteosarcoma [5, 6], suggesting MSCs a critical part of cancer stroma. MSCs, also known as mesenchymal stromal cells, are a heterogeneous group of multi-potent progenitor cells that could contribute to maintenance and regeneration of a variety of tissues [6, 7]. In the case of tissue injury or inflammatory, MSCs could be mobilized and recruited to the damage site upon sensing wound-associated signals [6]. Since cancer development is generally accompanied by multiple desmoplastic reactions, which confer the tumor site a ‘wound that never heals’ [8], MSCs has been reported to recruit to several cancer tissues, such as breast cancer [9], prostate cancer [10], and osteosarcoma [11]. And identified endocrine and paracrine signals, such as Sdf-1/CXCR4 and PGF/VEGFR1 axes [9], are found to be involved in this process. Therefore, previously studies mostly focused on the potential use of MSCs as vehicles for delivering anti-cancer agents. Till now, there are at least four clinical studies elucidating modulated-MSC-based therapy in patients with cancer but no solid results have yet been reported (ClinicalTrials.gov Identifier: NCT02008539, NCT02530047, NCT02068794, and NCT01983709).

However, MSCs could participate in tumor progression directly by influencing cancer cell biology or indirectly by modulating the immune status and angiogenic process, resulting the role MSCs played in tumor progression is complicated, especially in tumor metastasis [8]. Until recently, there are several studies focused on the effects of MSCs in tumor metastasis in animal models, but the results are conflicting. For example, Kaenoub et al. reported that MSCs within tumor stroma promote breast cancer metastasis [2], whereas Meleshina et al. found MSCs reduced metastasis of breast cancer [12]. Yan and his colleagues suggested opposite conclusion to Li and his colleagues’ conclusion that MSCs inhibit hepatocarcinoma metastasis [5, 13]. Moreover, to our knowledge, these relevant preclinical studies have never been systematically analyzed and strong evidence with animal model study is still lacking.

Therefore, in this review, we examine the current state-of-the-art of preclinical studies of the effect of MSCs administration on tumor metastasis, and we discuss their advantages, limitations and future potential.

## Methods

### Search strategy and literature selection

**Fig. 1:**
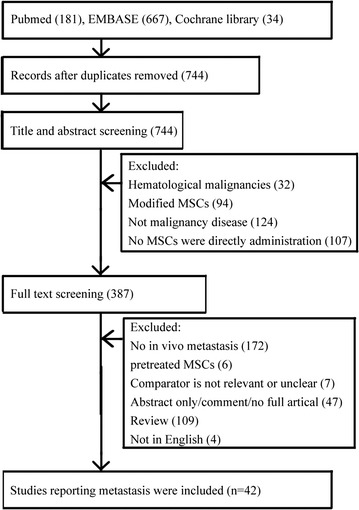
Flow diagram of the current meta-analysis. Studies of MSCs administration in animal models of solid tumor metastasis were identified from PubMed, EMBASE, and Cochrane library until March 2017. A total of 42 publications met the inclusion criteria

The PRISMA guidelines were used to conduct this review and meta-analysis [14]. Studies of MSCs administration in animal models of solid tumor metastasis were identified from PubMed, EMBASE, and Cochrane library from January 2000 to March 2017. The following search strategy was used for PubMed and EMBASE: (“mesenchymal stromal cell” OR “mesenchymal stem cell”) AND (cancer OR tumor) AND (preclinical OR animal) AND (metastasis OR progression). The search strategy used for Cochrane library was (mesenchymal stromal cell OR mesenchymal stem cell) AND (cancer OR tumor). Secondary references were also reviewed. Studies met all following criteria were included: (1) the study assessed effects of MSCs administration on incidence or number of metastasis in animal models with experimental cancer, (2) the research was performed in animals in vivo, (3) the research was an original full-text literature with unique data, (4) the study had appropriate control groups. Studies were excluded if the MSCs used involved additional active components such as gene/drug modification, or the cancer model was hematological malignancies rather than solid tumor. All the publications were randomly allocated to three independent reviewers (Li, Fan, and Wang), who screened out candidate papers mainly based on title and abstract. Full articles of candidate papers were subsequently analyzed in detail. The flow diagram of search strategy and literature selection is shown in Fig. 1.

### Data extraction

Data on animal model characteristics (animal species/strain and gender), cancer model (cancer cell type and implantation route), MSCs administration characteristics (MSCs origin, source, identification test, as well as administration route, dose, and timing related to cancer implantation), and primary outcome measures (region of metastasis, number or incidence of metastasis, and conclusion) were extracted. We also extracted bibliographic data, including authors, year of publication, and country or region.

For included 42 literatures, all independent comparisons of metastasis in animals with experimental cancer having MSCs or blank control treatment were identified. Replications were also collected separately. Information on primary outcome were extracted from both text and graphs, when raw data or mean/median/incidence, SD/SE were reported or recalculated. ImagePro Plus v6.0 software was used to obtain values from graphs. When the number of animals was reported as a range, the lowest group size was collected. When no clear data could be extracted, the report was excluded from further meta-analysis.

### Assessment of study quality and risk of bias

Quality and risk of bias was assessed by SYRCLE Risk of Bias tool [15]. When the number of animal per group reported in the Method section equaled to the number mentioned in the Results section or figure legends, we assumed there had no exclusion of animals. All the studies were reviewed independently by two reviewers.

Besides, to overcome the fact that there were too many items as “unclear” because of the poor description of details on experiment design and methods, we included 3 items in other Bias: (1) inappropriate influence of funders, (2) mention of randomization at any level, and (3) mention of blinding at any level. For inappropriate influence of funders, “Yes” indicated non-industry source of funding, no funding, or no conflict of interest, “No” indicated the study was funded by industry or author mentioned conflict of interests, “unclear” indicated funding source or conflict of interest was not mentioned. For mention of randomization or blinding, “Yes” indicated reported and “No” indicated unreported.

### Statistical analyses

Data were analyzed using Review Manager Software (RevMan, version 5.2). For the measurement of outcome event “number of metastases”, the standardized mean difference (SMD) was computed using mean and SD. If the data were shown as median and percentiles, the data were converted to mean and SD. For the measurement of outcome event “incidence of metastases”, the risk ration (RR) was computed. When individual comparisons with zero events in one group, either control or treatment group, a continuity correction of 0.5 was added to each cell, as implemented in RevMan software.

Considering the anticipated heterogeneity, random effects models were used to conducted meta-analysis. Mean effect size, 95% confidence intervals (95% CI), significance, and forest plots were analyzed by the inverse-variance method and the standard mean differences in RevMan software. The possibility of publication bias was assessed by visually evaluating the asymmetry in the funnel plots. Heterogeneity was examined by using I^2^.

Sensitivity analysis and subgroup analysis were performed to further assess the robustness of our findings and explain the observed heterogeneity

## Results

### Study selection process and study characteristics

As shown in Fig. 1, a total of 42 publications were identified. The detail characteristics of these studies were listed in Additional file 1: Table S1. Out of the 42 publications, 32 studies came with the conclusions that MSCs administration promoted outcome events (numbers or incidences of cancer metastasis) in animal studies. Excluded 3 publications that could not extract clear data, a total of 39 studies with 66 independent comparisons were included for further meta-analysis.

The characteristics of the included 39 publications with 66 independent comparisons were shown in Table 1. Breast cancer metastases were studies in 48.5% of the experiments. Metastases of osteosarcoma, hepatocellular carcinoma, colon cancer, and melanoma were studies in 12.1, 9.1, 6.1, and 4.5% of the experiments, respectively. The most popular metastases region was lung (62.1%), and the second was liver (10.6%). The remaining of 27.3% experiments studied the metastases in bone, lymph node, skin, or whole body in general.

**Table 1:** Characteristics of reviewed 39 publications with 66 comparisons (number = 66)

Characteristics	Subgroups	Number (%)
Cancer type	Breast cancer [2, 9, 12, 17, 18, 21, 32–40]	32 (48.5)
	Osteosarcoma [11, 19, 41–43]	8 (12.1)
	Hepatocellular carcinoma [5, 13, 44]	6 (9.1)
	Colon cancer [45–47]	4 (6.1)
	Melanoma [20, 48, 49]	3 (4.5)
	Other [10, 50–58]	13 (19.7)
Animal species/strain	Nude mice [2, 5, 9–13, 18, 19, 21, 32–40, 43–47, 50–54, 56–58]	55 (83.3)
	Other mice [17, 20, 48, 49, 55]	6 (9.1)
	Rat [41, 42]	5 (7.6)
Animal gender	Female [2, 12, 17, 18, 20, 33–37, 39, 40, 43, 46, 47, 50, 51, 53, 55, 56]	35 (53.0)
	Male [5, 10, 11, 19, 42, 45, 49, 54]	12 (18.2)
	Unclear [9, 13, 21, 32, 38, 41, 44, 48, 52, 57, 58]	19 (28.8)
Source of MSCs	Human bone marrow [2, 9–13, 18, 21, 32, 34, 35, 43–47, 52, 54, 56]	30 (45.5)
	Other human tissue [5, 33, 36–38, 50, 53, 57]	16 (24.2)
	Mice bone marrow [20, 35, 39, 40, 48, 49, 55, 58]	11 (16.7)
	Rat mone marrow [41, 42, 51]	6 (9.1)
	Unclear [17, 19]	3 (4.5)
Cancer cell/MSCs dose ratio	< 1 [2, 20, 33, 39, 46, 47, 49, 54, 55]	15 (22.7)
	=1 [5, 9, 12, 13, 17, 19, 21, 32, 36, 40–42, 44, 45, 48, 50, 51, 53, 57, 58]	32 (48.5)
	> 1 [10, 11, 18, 34, 35, 37, 38, 43, 52, 56]	19 (28.8)
Timing of MSCs administration	Co-administration [2, 5, 9, 10, 17–19, 21, 32–34, 36, 38–43, 45–48, 50, 52–55, 58]	47 (71.2)
	Followed administration [11–13, 20, 35–37, 41, 44, 49, 51, 56]	19 (28.8)
MSCs passages	Reported (3–20) [2, 12, 13, 19, 20, 32, 35, 37, 42, 44, 49–52]	25 (37.9)
	Unclear [5, 9–11, 17, 18, 21, 33, 34, 36, 38–41, 43, 45–48, 53–58]	41 (62.1)
MSCs differentiation	With [18, 20, 21, 32, 33, 35, 39, 43, 45, 46, 48, 50, 52, 53, 56, 57]	25 (37.9)
	Without [2, 5, 9–13, 17, 19, 34, 36–38, 40–42, 44, 47, 49, 51, 54, 55, 58]	41 (62.1)
Metastasis site	Lung [2, 5, 9, 11–13, 17–21, 32, 33, 35–40, 42–45, 49, 51, 54, 56, 58]	41 (62.1)
	Liver [18, 33, 35, 46, 47, 52, 57]	7 (10.6)
	Other organ or multiple organ [9, 10, 17, 18, 32, 34, 48, 53, 55]	18 (27.3)

Out of the studies, most experiments (83.3%) conducted in nude mice. Female and male animals were used in 53.0% and 18.2% of the studies. MSCs derived from human bone marrow were used in most experiments (45.5%). Also, MSCs derived from human umbilical cords blood or adipose tissues were used in 24.2% of the studies. Besides, one study included MSCs derived from primary human pancreatic cancer tissues. The ratio of tumor cells to MSCs varied between 0.01 and 50, while the number of administrated MSCs was consistent with the number of injected tumor cells in most experiments (48.5%). MSCs were generally administrated as a single does together with tumor cells, and in 22.7% experiments animals received multiple MSCs injections via tail vein within 5 weeks following tumor cells injections.

Out of the 39 studies, only 14 of them reported the passages of MSCs they used, and 16 of them identified MSCs by verified the potentials of multilineage differentiation, with most studies failed to report the passages and differentiation potentials of the MSCs used.

### Study quality and risk of bias

**Fig. 2:**
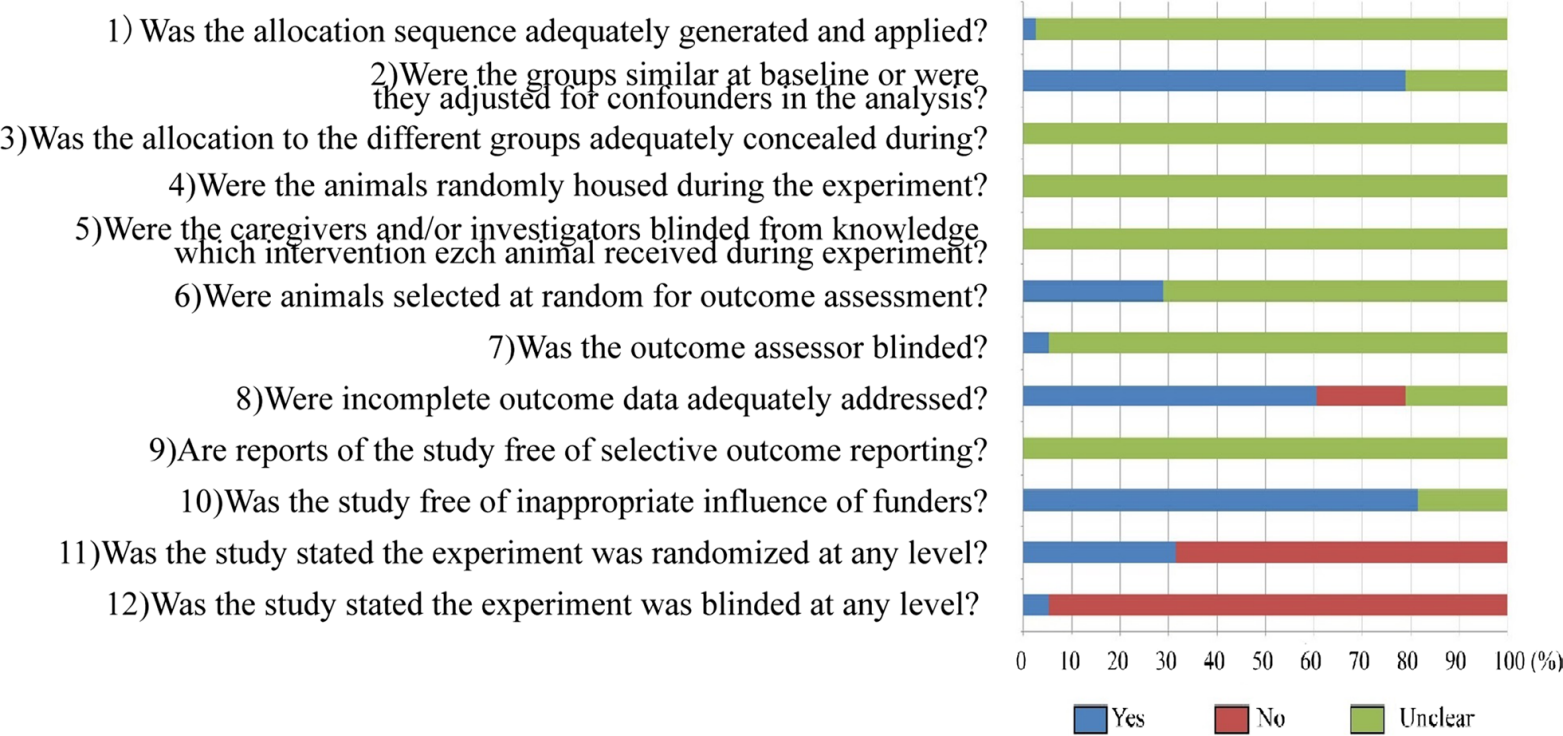
Proportion of studies meeting each quality score criterion. Results of the risk of bias of the 39 studies involved in the systematic review were shown by bar charts. For the items 1 to 10, “Yes” means that the description of measurements meets the item requirements, indicating low risk of bias, “No” means that the description does not meet the item requirements, indicating high risk of bias, and “Unclear” means there is no relevant reports, indicating unclear risk of bias. With the item 11 and 12, “Yes” indicated reported and “No” indicated unreported

The quality assessment of the 39 studies was shown in Fig. 2. In most cases, poor reporting resulted in an unclear risk of bias. Take selection bias as an example, almost no author described the randomization procedures or whether the sequence had been concealed. However, the majority of studies seemed to be similar at animal baseline. Performance bias was reflected by item 4 and 5. As shown in Fig. 2, none of the authors reported the measures to reduce the performance bias. Measures to reduce detection bias (item 6 and 7) were mentioned in a small part of studies, though the specific methods to achieve randomization for outcome assessment were not reported. Concerning the risk of attrition bias (item 8), most studies were low risk of bias, while 18.4% high risk and 21.1% unclear. Date to assess the risk of reporting bias (item 9) were incomplete in studies included, leading to an unclear risk of bias.

Considering the fact that only limited details on most animals’ studies were provided, we scored another three items as described in the method section. As shown in Fig. 2, most studies were low risk of inappropriate fund bias (81.6%). Randomization of the studies at any level was reported in 31.6% publications. And blinding at any level was reported in 5.3% publications.

According to whether the publication meets the 12 items listed above, we classify the publications into three categories. Among all the 39 publications, no study has reached 9–12 criteria, only seven publications have met 5–8 criteria, and 32 publications have met 0–4 criteria.

### Meta-analysis of the effect size: incidence of tumor metastases

**Fig. 3:**
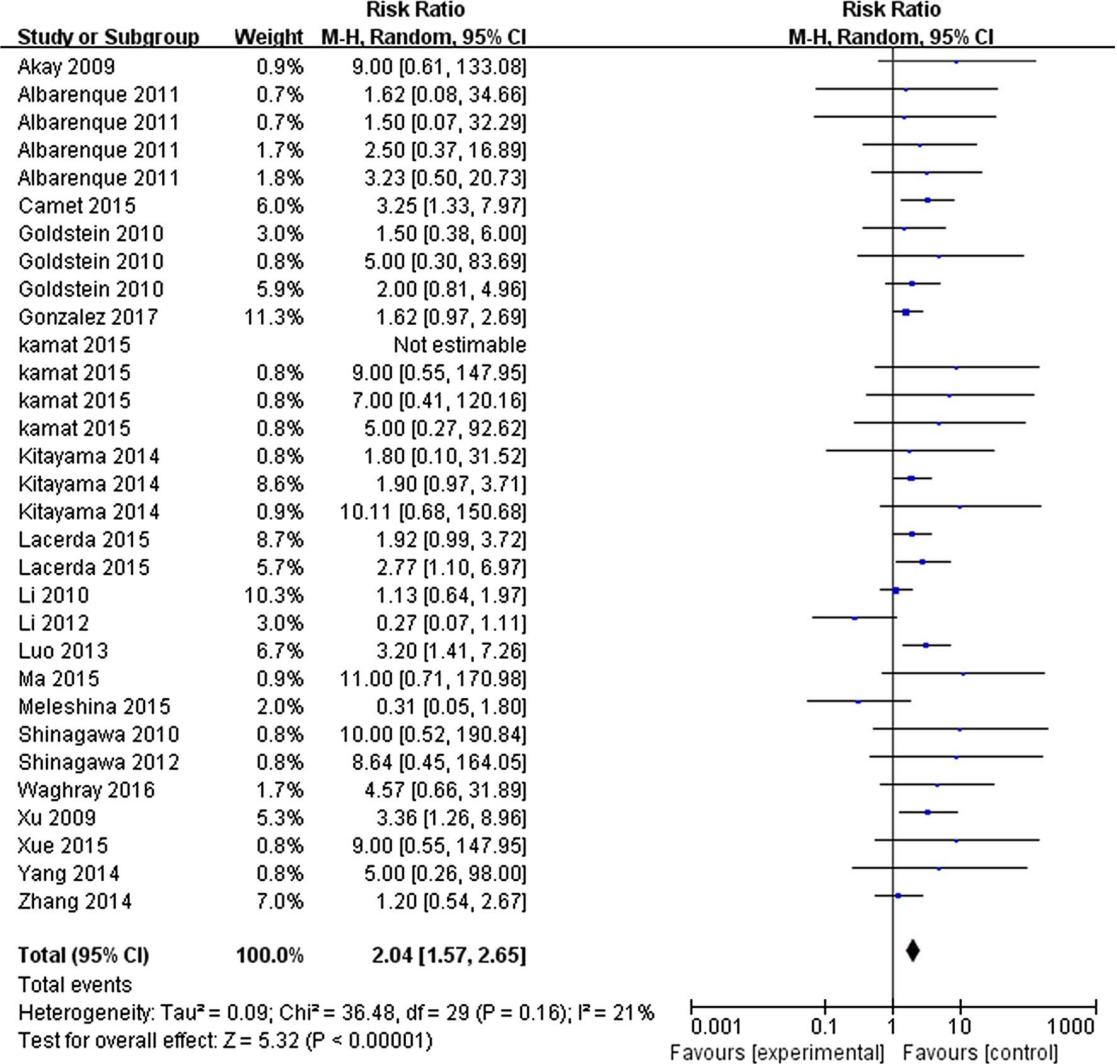
Forest plots of meta-analysis for the association between MSC administration and the incidence of tumor metastasis in animal models. A total of 31 comparisons with 624 animals were involved. It found the administration of MSCs increased the incidence of tumor metastases in animal models (RR 2.04, 95% CI 1.57–2.65, I^2^ = 21%)

A total of 31 comparisons with 624 animals investigating the effect of MSCs administration on the incidence of tumor metastases in experimental animal models were involved in the meta-analysis. Overall, the results found the administration of MSCs increased the incidence of tumor metastases in animal models (RR 2.04, and 95% CI 1.57–2.65, Fig. 3), with a mild heterogeneity between studies (I^2^ = 21%).

Subgroup analyses were performed based on experiment design, to examine whether particular factors may affect the outcome of meta-analysis. The effects of MSCs on the incidence of cancer metastasis were robust across cancer type, cancer metastasis site, animal models, as well as MSCs delivery timing, source, and dose (Table 2). Also, no significant impact of quality assessment category on effect size was observed (*p *= 0.63). Besides, considering that the quality and risk of bias due to poor reporting in most cases was not clear, experiments were also grouped according to the impact factors (IFs) of the journal. And it found the effects were robust across these two groups (*p *= 0.58).

**Table 2:** Effect size and subgroup analyses for MSC administration in preclinical studies of solid tumor metastasis incidence

Group	Weight	Effect size		I^2a^	*P*	I^2b^
	(%)	RR	95% CI	(%)		(%)
All experiments	100.0	2.04	1.57, 2.65	21		
Cancer type						
Breast cancer	45.8	1.94	1.43, 2.62	0	0.630	0
Other cancer	54.2	2.22	1.41, 3.50	21		
Animal species/strain						
Nude mice	86.2	2.03	1.53, 2.68	19	0.810	0
Other mice/rat	13.8	2.30	0.85, 6.22	54		
Source of MSC						
Human bone marrow	65.1	1.81	1.24, 2.64	42	0.450	0
Other human tissue	16.1	2.77	1.61, 4.77	0		
Mice/rat bone marrow	18.8	2.09	1.24, 3.51	0		
Metastasis estimation						
Fluorescence microscopy	17.2	2.95	1.32, 6.60	27	0.610	0
Histological evaluation	34.6	1.79	1.03, 3.14	38		
Bioluminescence	48.2	2.13	1.59, 2.84	0		
Cancer cell/MSC dose ratio						
= 1	47.2	1.39	0.9, 2.16	38	0.030	71.6
> 1	45.3	2.62	1.89, 3.65	0		
< 1	7.5	3.83	1.68, 8.73	0		
Timing of MSC administration						
Co-administration	73.6	2.19	1.72, 2.79	0	0.240	28.5
Follow-administration	26.4	1.39	0.68, 2.84	48		
Study length (days)						
≤ 42	100	2.04	1.57,2.65	21	–	–
> 42	0	–	–	–		
Metastasis site						
Lung	55.4	1.61	1.08, 2.40	34	0.060	72
Other tissue	44.6	2.64	1.92, 3.63	0		
Study quality category						
Meet 9–12 criteria	0	–	–	–	0.630	0
Meet 5–8 criteria	9.2	2.90	0.74, 11.40	37		
Meet 0–4 criteria	90.8	2.06	1.56, 2.71	21		
Impact factor of journal						
≤ 5	49.3	1.90	1.19, 3.01	36%	0.580	0
> 5	50.7	2.21	1.66, 2.95	0		

*RR* risk ratio; *CI* confidence interval; *I*^*2a*^*, I*^*2*^ for heterogeneity within each subgroup; *P P* value for heterogeneity between subgroups; *I*^*2b*^*, I*^*2*^ for heterogeneity between subgroups

Heterogeneity within the subgroup was remarkably reduced when cancer type, metastasis estimation methods, MSCs administration way, and IFs were limited. For example, subgroup analysis for breast cancer, which weighted 45.8%, found an effect size RR 1.94 (95% CI 1.43–2.62, I^2^ = 0%). Subgroup analysis for bioluminescence measurement of metastasis, which weighted 48.2%, found an effect size RR 2.13 (95% CI 1.59–2.84, I^2^ = 0%). Subgroup of co-administration of cancer cells and MSC, weighted 73.6%, found an effect size RR 2.19 (95% CI 1.72–2.79, I^2^ = 0%), and subgroup of higher IFs, weighted 50.7%, found an effect size RR 2.21 (95% CI 1.66–2.95, I^2^ = 0%). What’s more, it shown cancer cells/MSCs injection ratio may be the main source of heterogeneity (*p *= 0.03, heterogeneity between subgroups I^2^ = 71.6%). However, because the number of studies in the subgroup of cancer cells/MSCs ratio <1 was small, the results should be interpreted with cautions.

### Meta-analysis of the effect size: number of tumor metastases

**Fig. 4:**
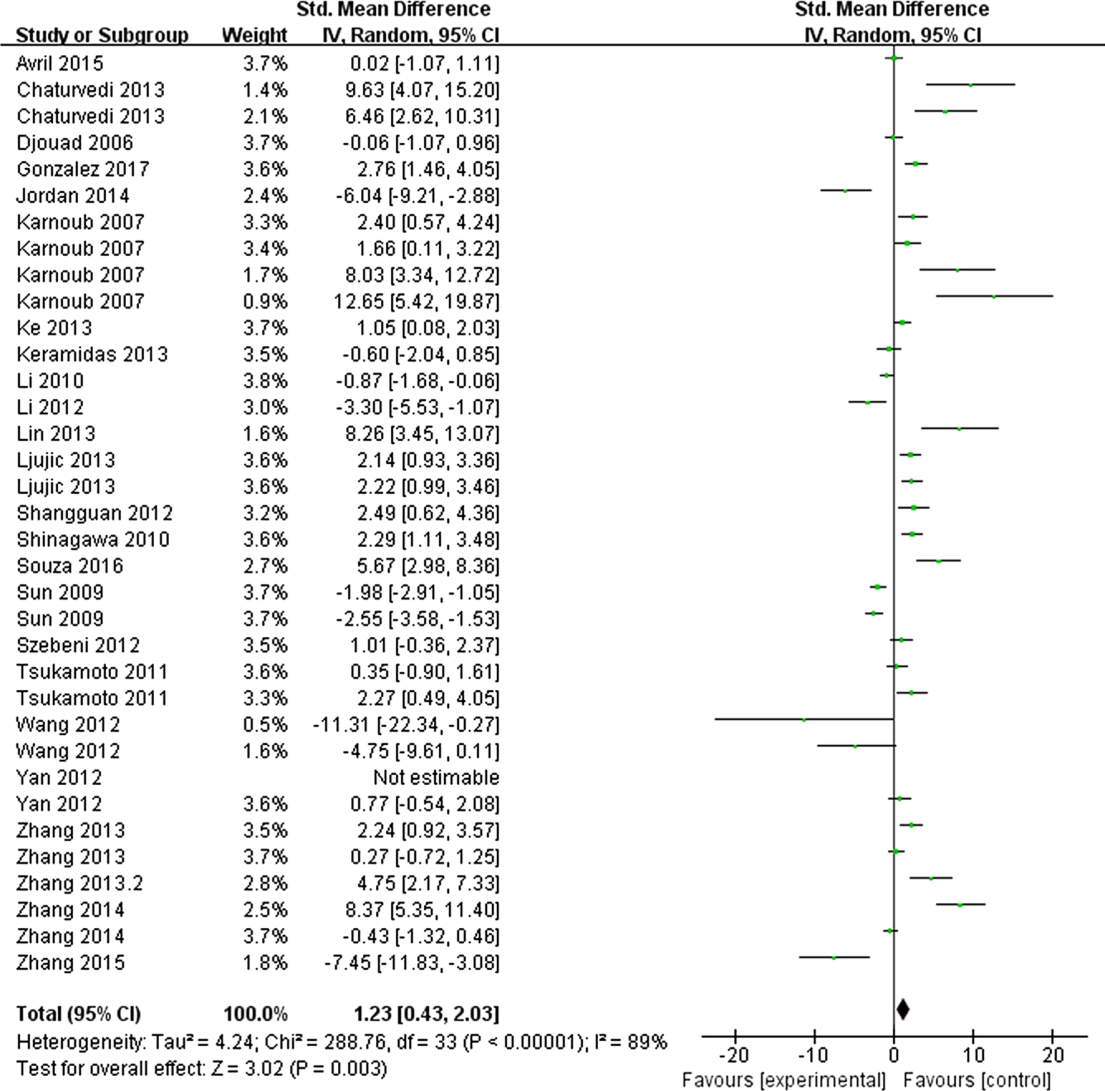
Forest plots of meta-analysis for the association between MSC administration and the number tumor metastasis in animal models. A total of 35 comparisons with 489 animals were involved. It found the administration of MSCs increased the number of tumor metastases in experimental cancer models (SMD 1.23, 95% CI 0.43–2.03, I^2^ = 89%)

A total of 35 comparisons with 489 animals were involved in the meta-analysis, which investigating the effect of MSCs administration on the number of tumor metastases. The results found the administration of MSCs increased the number of tumor metastases in experimental cancer models (SMD 1.23, and 95% CI 0.43–2.03, Fig. 4). The heterogeneity between studies was significant (I^2^ = 89%), demonstrating notable heterogeneity across studies.

Subgroup analyses were shown in Table 3. It revealed the effects varied across the way MSCs administrated (both dose and timing), the study length, and impact factors of published journal. But no significant impact of quality assessment category on effect size was observed (*p *= 0.07). Subgroups of co-administration of cancer cells and MSCs, study length longer than 42 days, and journals had higher impact factors (IF>5) had reduced heterogeneity.

**Table 3:** Effect size and subgroup analyses for MSC administration in preclinical studies of solid tumor metastasis number

Group	Weight	Effect size		I^2a^	*P*	I^2b^
	(%)	SMD	95% CI	(%)		(%)
All experiments	100.0	1.23	0.43, 2.03	89		
Cancer type						
Breast cancer	48.6	2.04	0.87, 3.21	90	0.060	71.8
Other cancer	51.4	0.48	− 0.65, 1.61	89		
Animal species/strain						
Nude mice	82.3	1.12	0.26, 1.97	89	0.690	0
Other mice/rat	17.7	1.67	− 0.9, 4.23	92		
Source of MSC						
Human bone marrow	40.7	1.96	0.59, 3.33	87	0.330	12.4
Other human tissue	18.2	0.09	− 1.94, 2.13	94		
Mice/rat bone marrow	31.0	0.73	− 0.72, 2.17	89		
Unclear	10.0	2.15	− 0.14, 4.34	85		
Metastasis estimation						
Fluorescence microscopy	42.0	1.11	− 0.3, 2.35	90	0.170	44.2
Histological evaluation	40.1	2.02	0.76, 3.29	88		
Bioluminescence	17.9	− 0.54	− 2.95, 1.87	89		
Cancer cell/MSC dose ratio						
= 1	62.6	1.11	0.1, 2.12	90	0.030	72.7
> 1	7.2	− 0.20	− 1.08, 0.67	0		
< 1	30.2	1.85	0.45, 3.24	80		
Timing of MSC administration						
Co-administration	68.5	2.18	1.38, 2.99	82	0.001	94
Follow-administration	31.5	− 1.13	− 2.50, 0.23	88		
Study length (days)						
≤ 42	59.1	0.32	− 0.70, 1.35	91	0.006	86.7
> 42	40.9	2.44	1.32, 3.56	78		
Metastasis site						
Lung	81.6	1.80	0.28, 2.07	89	0.570	0
Other tissue	18.4	1.66	0.26, 3.07	73		
Study quality category						
Meet 9–12 criteria	0	–	–	–	0.07	70.5
Meet 5–8 criteria	20.3	2.92	0.90, 4.94	90		
Meet 0–4 criteria	79.7	0.85	− 0.04, 1.74	89		
Impact factor of journal						
≤ 5	56.8	0.30	− 0.73, 1.32	89	0.004	87.7
> 5	43.2	2.30	1.38, 3.22	74		

*SMD* standard mean difference; *CI* confidence interval; *I*^*2a*^*, I*^*2*^ for heterogeneity within each subgroup; *P P* value for heterogeneity between subgroups; *I*^*2b*^*, I*^*2*^ for heterogeneity between subgroups

To rule out the influence of cancer type on heterogeneity, experiments of breast cancer were selected and further subgroup analyses were performed. It also revealed study length and different ways of metastasis measurement or MSCs administration could be the main sources of heterogeneity (Table 4). For example, subgroup analysis for histological measurement of metastasis found an effect size SMD 2.92 (95% CI 1.82–4.02, I^2^ = 55%). Whereas subgroup for fluorescence measurement found an effect size SMD 1.22 (95% CI − 0.2~2.65, I^2^ = 90%).

**Table 4:** Effect size and subgroup analyses for MSC administration in preclinical studies of breast cancer metastasis number

Group	Weight	Effect size		I^2a^	*P*	I^2b^
	(%)	SMD	95% CI	(%)		(%)
All experiments	100.0	2.04	0.87, 3.21	90		
Animal species/strain						
Nude mice	100	2.04	0.87, 3.21	90	–	–
Other mice/rat	0	–	–	–		
Source of MSC						
Human bone marrow	40.3	4.06	2.43, 5.70	70	0.006	75.8
Other human tissue	30	− 0.07	− 2.54, 2.41	95		
Mice/rat bone marrow	14.8	1.04	0.24, 1.83	0		
Unclear	14.9	1.20	− 0.73, 3.14	82		
Metastasis estimation						
Fluorescence microscopy	64.1	1.22	− 0.2, 2.65	90	0.07	70.5
Histological evaluation	35.9	2.92	1.82, 4.02	55		
Bioluminescence	0	–	–	–		
Cancer cell/MSC dose ratio						
= 1	58.8	1.48	− 0.14, 3.1	92	0.35	0
> 1	0	–	–	–		
< 1	41.2	2.44	1.27, 3.62	65		
Timing of MSC administration						
Co-administration	84.8	2.38	1.54, 3.22	72	0.001	98
Follow-administration	15.2	− 2.24	− 2.93, 1.55	0		
Study length (days)						
≤ 42	44.8	0.29	− 1.42, 2.00	93	0.008	85.7
> 42	55.2	3.24	1.88, 4.6	78		
Metastasis site						
Lung	75.6	1.80	0.47, 3.12	90	0.680	0
Other tissue	24.4	2.20	0.84, 3.56	60		
Impact factor of journal						
≤ 5	30	− 0.64	− 2.52, 1.23	92	0.002	90
> 5	70	2.8	1.78, 3.82	73		

*SMD* standard mean difference; *CI* confidence interval; *I*^*2a*^*, I*^*2*^ for heterogeneity within each subgroup; *P P* value for heterogeneity between subgroups; *I*^*2b*^*, I*^*2*^ for heterogeneity between subgroups

### Sensitivity analysis

To assess the robustness of the estimated pooled effect size for metastases number and incidence, we performed a leave-one-out sensitivity analysis by removing one study at a time and reevaluating the effect size of the remaining studies. For both the number and the incidence of metastases, the pooled effect was stable, indicating the results were not driven by any single study.

### Publication bias

**Fig. 5:**
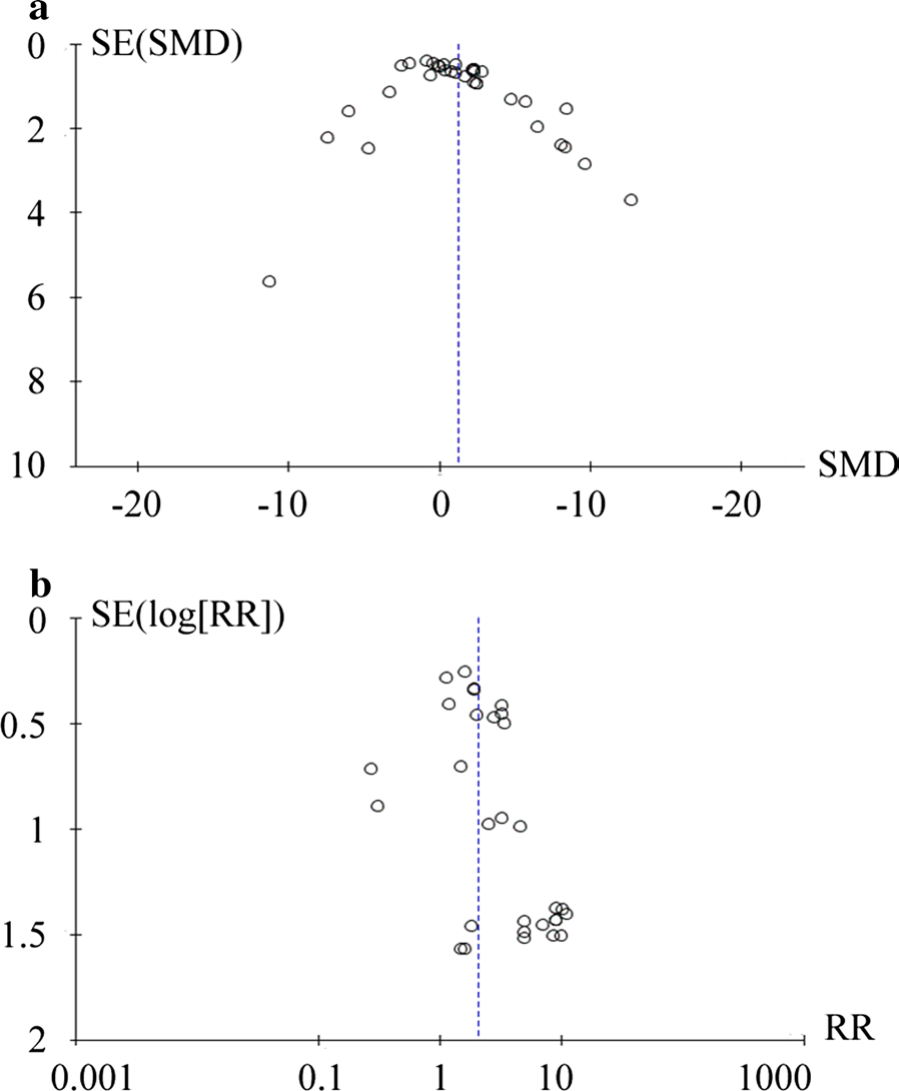
Funnel plots showed the distribution of researched study outcomes to estimate potential publication bias. **a** Number of metastasis; **b** Incidence of metastasis

Publication bias was assessed by funnel plot for the outcomes of metastases number and incidence. Figure 5a suggested a symmetrical distribution for the number of metastases. But funnel plot demonstrated some degree of asymmetry for the incidence of metastases, indicating the possibility of either publication bias or a systematic difference between the studies (Fig. 5b).

## Discussion

It has been reported that there might be a relation between MSCs and the outgrowth of cancer metastasis [5, 16–18]. Solid preclinical analysis, however, has been lacking so far. The current meta-analysis examined the effect of MSCs on cancer metastasis in current state-of-the-art preclinical studies. We provide evidence that MSCs usage increase the number and the incidence of cancer metastasis in experimental cancer models.

To our knowledge, no studies have examined the effects of MSCs on solid tumor metastasis in animal models using a meta-analysis. Our meta-analysis suggested MSCs increased the number and the incidence of tumor metastases in animal models (SMD 1.23, 95% CI 0.43–2.03; RR 2.04, 95% CI 1.57–2.65, respectively). These studies advanced our understanding of the pro-metastasis properties of MSCs during tumor metastasis. More specifically, as shown in Additional file 2: Figure S1, MSCs could produce large amounts of factors and exosomes to promote cancer cell invasion, angiogenic process, or prepare tissue niches in distant organs [6, 19]. MSCs could also affect cancer cells by cell–cell contact. Moreover, MSCs could recruit several immunosuppressive stroma cells such as myeloid-derived suppressor cells (MDSCs) by creating chemokines [20]. Notably, these pro-metastasis properties of MSCs might transiently expressed in response to context signals, such as hypoxia in tumor niches, rather than constitutively expressed [9, 21, 22]. However, as cell lines study has its intrinsic limitation, the conclusions and underlying mechanisms should be explained with cautions. And it is important to implement studies on primary biopsy material to demonstrate the effect of MSCs.

In general, an effect size of 0.8 equates to a large effect, 0.5 to a medium effect, and 0.2 to a small effect [23]. In this context, the effects of MSCs on cancer metastasis can be classified as large, for example, for number of metastasis averaging 1.23 and incidence of metastasis averaging 2.04 across all comparisons. In this study, effects of MSCs on the incidence of cancer metastasis were robust across cancer type, cancer metastasis site, animal models, as well as MSCs delivery timing, source, and dose (Table 2).

The quality of the studies was also reviewed, using SYRCLE’s risk of bias tool for animal studies [15]. We found that the risk of bias could not be estimated in most studies for each item assessed, indicating a poor reported risk of bias. Only seven publications have met more than four items out of the 12 criteria. Though no significant impact of quality assessment category on effect size was observed (*p *= 0.07 for the number of cancer metastasis and *p *= 0.63 for the incidence of cancer metastasis), and current reporting quality of pre-clinical studies is generally poor, it may be associated with inflations in the estimates of the effect size [24–27]. Moreover, methodological weakness in animal studies could result in over-estimated treatment effects and decrease confidence in translational potential [25]. Thus, further pre-clinical studies with high reporting quality of essential experimental details is needed.

Besides, heterogeneity was observed in current meta-analyses. On one hand, based on this review, the level of heterogeneity between the studies for the incidence of cancer metastasis was low (I^2^ = 21%), suggesting that incidence of metastasis might be an efficient indicator of effect of MSCs on cancer metastasis. On the other hand, the level of heterogeneity between the studies for the number of cancer metastasis was relatively high (I^2^ = 89%), though the I^2^ values were similar to the values reported by other studies focused on animal studies [24, 28, 29]. Further subgroup analysis, to some extent, revealed some possibilities of heterogeneity, such as study length, methods of metastasis measurement and MSCs administration. We observed that short-term study length (≤ 42 days), metastasis count by fluorescence intensity rather than histological measurement, and MSCs intravenously injected more than once after primary tumor implanted may induce high heterogeneity. To some extent, this might help to inform the design of future animal studies.

Since MSCs are a heterogeneous population of cells, it is relevant to view the source of MSCs and the techniques used to isolate and characterize MSCs in original studies [6, 7, 30, 31]. Among all the 39 studies involved in present meta-analysis, however, only 16 studies reported on multi-lineage differentiation for MSCs and 14 studies reported on cell passages of MSCs used. Also, these studies used different sets of molecular or phenotypic pattern to characterize MSCs. These differences in cell isolation and identification might favor certain subpopulations, which could result in the heterogeneity. Thus, further studies should pay more attention to the methods which clarified the population of stromal cells that used experimentally, to enable adequate analysis across different studies.

Finally, this review has several limitations. First, we could not rule out that the effect of MSCs could be less strong or not effective in certain subgroups of unpublished data, since the visualization of the funnel plot suggested some extent of publication bias. Second, the level of heterogeneity between the studies for the number of cancer metastasis was high, though random effects models were used to account for anticipated heterogeneity. Thus the substantial heterogeneity found through this review may restrict the generalizability of the findings. Third, there were only 39 publications met the inclusion criteria, though 744 literatures were identified by electronic searching. Nevertheless, at the very least our analysis represents the complete review for unmodified-MSCs administration in experimental cancer metastasis.

## Conclusion

The present meta-analysis demonstrated the favorable impact of MSCs on cancer metastasis, both the incidence and the number, in experimental cancer models. Although poor reported risk of bias and high level of heterogeneity were yet limited, at the very least, the current meta-analysis underlined that MSCs might have an active role in tumor microenvironment and might represent a promising target to therapies preventing the establishment of cancer distant metastasis. Still further preclinical studies with better design and adequate reporting are needed.

## Additional files

**Additional file 1: Table S1.** The detail characteristics of all the publications included.

**Additional file 2: Figure S1.** MSCs with promoting effects on tumor metastasis.

## Abbreviations

MSCs: mesenchymal stem cells
SMD: standardized mean difference
RR: risk ration
95% CI: 95% confidence intervals
MDSCs: myeloid-derived suppressor cells
