# Supplementary material for: Effects of mesenchymal stem cells on solid tumor metastasis in experimental cancer models: a systematic review and meta-analysis
Source: J Transl Med. 2018 Apr 27;16:113. doi: 10.1186/s12967-018-1484-9 (PMC5924448; doi:10.1186/s12967-018-1484-9)
Supplement: Supplementary file 2 — Additional file 2: Figure S1. MSCs with promoting effects on tumor metastasis. [file 12967_2018_1484_MOESM2_ESM.docx]

Supplementary figure 1: MSCs with promoting effects on tumor metastasis.


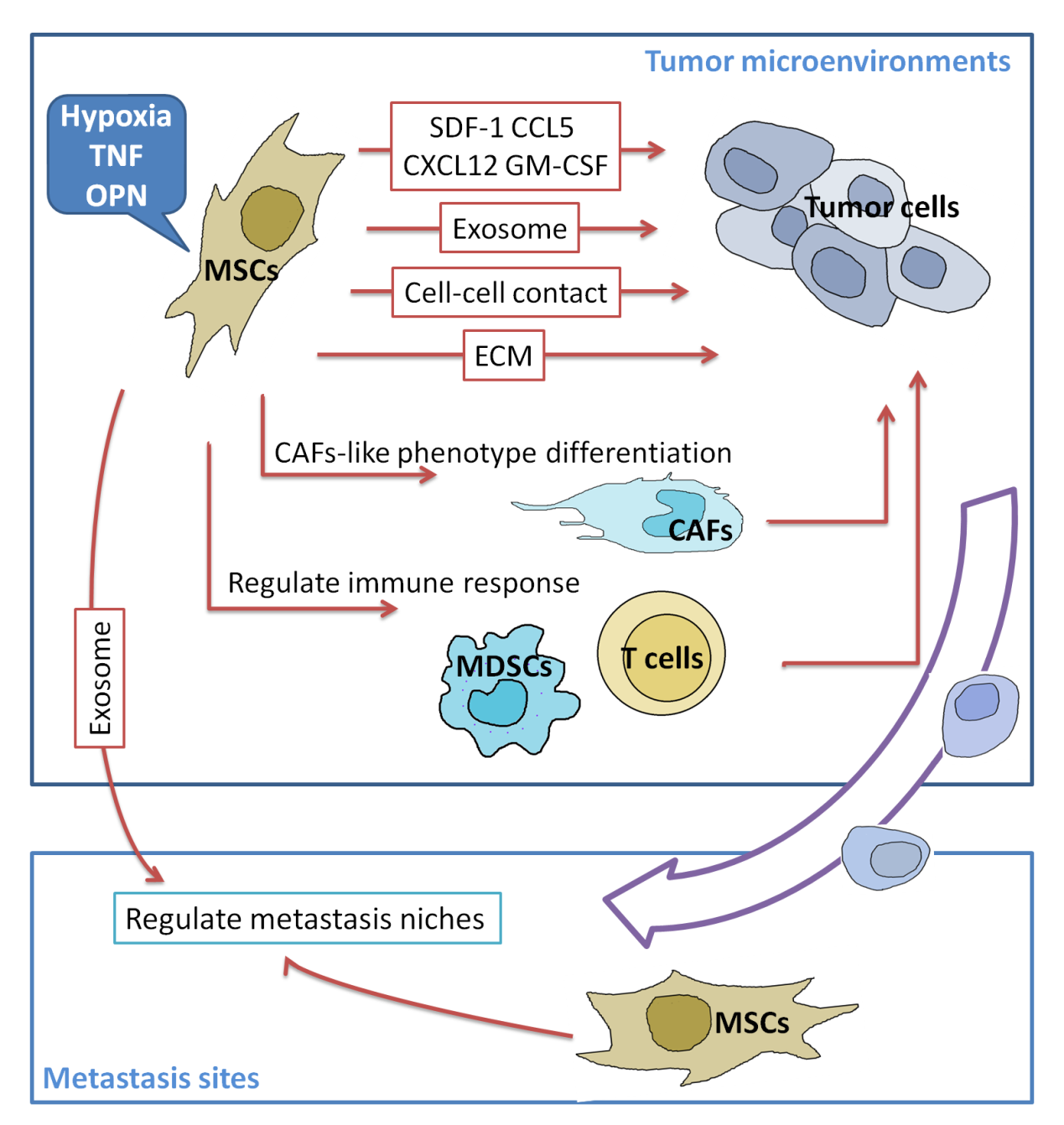


MSCs produce several pro-metastatic factors, such SDF-1, CCL5, CXCL12, and so on [1,2]. MSCs also affect tumor cells by producing CD81-positive exosome, which could contain mRNAs and proteins [3]. MSCs could directly interact with tumor cells and enhance the expression of miR-199a and promoting invasive properties. MSCs secrete extracellular matrix, such as collagen, and regulate tumor microenvironments (TMEs) [4]. MSCs could acquire the carcinoma-associated fibroblasts (CAFs)-like phenotype and promote tumor metastasis [5]. MSCs modulate immunosuppressive niche by facilitating the recruitment or function of myeloid-derived suppressor cells (MDSCs) or T cells [6,7]. Besides, TMEs could regulate the function of MSCs, such as the hypoxia in the tumor site, or cytokines secreted by tumor cells [8,9]. In addition, exosome from MSCs and MSCs recruited to metastasis sites help to prepare niches for the arrival of metastatic tumor cells [10,11].

Reference:

[1] Karnoub AE, Dash AB, Vo AP, Sullivan A, Brooks MW, Bell GW, et al. Mesenchymal stem cells within tumour stroma promote breast cancer metastasis. Nature 2007;449:557-63.

[2] Waghray M, Yalamanchili M, Dziubinski M, Zeinali M, Erkkinen M, Yang H, et al. GM-CSF Mediates Mesenchymal-Epithelial Cross-talk in Pancreatic Cancer. Cancer Discov 2016;6:886-99.

[3] Luga V, et al. Exosomes mediate stromal mobilization of autocrine wnt-PCP signaling in breast cancer cell migration. Cell 2012;151:1542-1556.

[4] Gonzalez ME, Martin EE, Anwar T, Arellano-Garcia C, Medhora N, Lama A, et al. Mesenchymal Stem Cell-Induced DDR2 Mediates Stromal-Breast Cancer Interactions and Metastasis Growth. Cell Rep 2017;18:1215-1228.

[5] Waghray M, Yalamanchili M, Dziubinski M, Zeinali M, Erkkinen M, Yang H, et al. GM-CSF Mediates Mesenchymal-Epithelial Cross-talk in Pancreatic Cancer. Cancer Discov 2016;6:886-99.

[6] Souza LE, Almeida DC, Yaochite JN, Covas DT, Fontes AM. Intravenous administration of bone marrow-derived multipotent mesenchymal stromal cells enhances the recruitment of CD11b(+) myeloid cells to the lungs and facilitates B16-F10 melanoma colonization. Exp Cell Res 2016;345:141-9.

[7] Ljujic B, Milovanovic M, Volarevic V, Murray B, Bugarski D, Przyborski S, et al. Human mesenchymal stem cells creating an immunosuppressive environment and promote breast cancer in mice. Sci Rep 2013;3:2298.

[8] Mi Z, Bhattacharya SD, Kim VM, Guo H, Talbot LJ, Kuo PC. Osteopontin promotes CCL5-mesenchymal stromal cell-mediated breast cancer metastasis. Carcinogenesis 2011;32:477-87.

[9] Chaturvedi P, Gilkes DM, Wong CC, Luo W, Zhang H, Wei H, et al. Hypoxia-inducible factor-dependent breast cancer-mesenchymal stem cell bidirectional signaling promotes metastasis. J Clin Invest 2013;123:189-205.

[10] Peinado H, et al. Melanoma exosomes educate bone marrow progenitor cells toward a pro-metastatic phenotype through MET. Nat Med 2012;18:883-891.

[11] Kaplan RN, et al. VEGFR1-positive haematopoietic bone marrow progenitors initiate the pre-metastatic niche. Nature 2005;438:820-827.
